# Associations of role, area deprivation index, and race with health behaviors and body mass index among localized prostate cancer patients and their partners

**DOI:** 10.1007/s11764-024-01625-z

**Published:** 2024-06-18

**Authors:** Jingle Xu, Chunxuan Ma, Rachel Hirschey, Jia Liu, Daria B. Neidre, Matthew E. Nielsen, Thomas C. Keyserling, Xianming Tan, Lixin Song

**Affiliations:** 1 School of Nursing, University of North Carolina at Chapel Hill, Chapel Hill, NC USA; 2 School of Nursing, The University of Texas Health Science Center at San Antonio, San Antonio, TX USA; 3 Lineberger Comprehensive Cancer Center, University of North Carolina at Chapel Hill, Chapel Hill, NC USA; 4 School of Medicine, University of North Carolina at Chapel Hill, Chapel Hill, NC USA; 5 Department of Biostatistics, Gillings School of Global Public Health, University of North Carolina at Chapel Hill, Chapel Hill, NC USA; 6 Mays Cancer Center, The University of Texas Health Science Center at San Antonio, San Antonio, TX USA

**Keywords:** Health behavior, Prostate cancer, Couple, Health inequities, Race, Area deprivation index

## Abstract

**Purpose:**

To examine the associations of role (localized prostate cancer (PCa) patient vs. their intimate partner), area deprivation index (ADI—higher scores indicating higher neighborhood deprivation levels), and race (Black/African American (AA) vs. White) with health behaviors and body mass index (BMI) among PCa patients and partners. The behaviors include smoking, alcohol consumption, diet quality, sedentary behaviors, and physical activity (PA).

**Methods:**

This study used the baseline data collected in a clinical trial. Given the nested structure of the dyadic data, multi-level models were used.

**Results:**

Significant role-race interaction effects on smoking, ADI-race effects on alcohol consumption, and role-ADI effects on BMI were found. Meanwhile, patients smoked more cigarettes, decreased alcohol consumption, had less healthful diets, spent longer time watching TV, did fewer sedentary hobbies, had more confidence in PA, and had higher BMIs than their partners. High ADI was independently associated with lower odds of drinking alcohol, using computer/Internet, and doing non-walking PA, and higher BMI compared to low ADI controlling for role and race. Black/AA dyads had less smoking amount and alcohol consumption and higher sedentary time and BMI than White dyads when adjusted for role and ADI.

**Conclusions:**

This study identified significant interaction and main effects of role, ADI, or race on health behaviors and BMI.

**Implications for Cancer Survivors:**

Future behavioral interventions should address divergent individual needs between patients and partners, social and neighborhood barriers, and cultural indicators of racial groups to promote healthful behaviors and improve the quality of survivorship for PCa patients and partners.

**Supplementary Information:**

The online version contains supplementary material available at 10.1007/s11764-024-01625-z.

Prostate cancer (PCa) is the most prevalent cancer among men in USA, excluding skin cancer, with its 5-year survival rate for localized stage reaching 100% [1]. More than 3.1 million men live beyond a PCa diagnosis in USA [2]. The American Cancer Society (ACS) recommends that individuals quit smoking, limit alcohol consumption, follow healthy eating patterns, stay physically active, and maintain a healthy body weight [3] to prevent cancer and improve survival outcomes. For both patients with localized PCa and their partners (i.e., their spouse or cohabiting partner), engaging in beneficial health behaviors mitigates their poor quality of life [3–5], as these health behaviors alleviate patients’ symptoms[6–8] and help manage their comorbid conditions [3, 9–12], and consequently relieve caregiver burden and psychological distress for their partners [13, 14]. Considering that PCa patients have low adherence to healthful behaviors [15–17] and their health behaviors are significantly correlated with their spouses’ [18], couple-based lifestyle behavioral interventions may effectively encourage PCa patients and partners to adhere to healthful behaviors and benefit their health conditions [19]. However, few couple-based behavioral interventions have explicitly focused on this population with limited effects [19–21]. To inform future couple-based health behavior interventions tailored to the needs of PCa patients and partners, this secondary data analysis investigates the effect of multi-level factors (role, area deprivation index (ADI), race) on their health behaviors.

Research has confirmed the positive correlations between PCa patients and partners in their fruit and vegetable intake and physical activity (PA) [18]; however, the differences between their health behaviors, potentially indicating their separate individual needs, have been understudied in the current literature. For example, partners, who are usually family caregivers to PCa patients, may not engage in healthful behaviors due to caregiving-related time constraints or mental distress [22, 23]. Understanding these differences will guide the development of tailored couple-based interventions that address the diverse needs and challenges PCa patients and partners face to improve the efficacy and sustainability of these interventions.

Most cancer-related behavioral research has focused on individual-level cognitive factors (e.g., self-efficacy, outcome expectations, and goal-setting) [24, 25]; little research has examined how one’s social and neighborhood context (e.g., educational access and economic stability) affects their health behaviors [26, 27]. For example, a highly deprived neighborhood may have limited education resources, unsupportive environments for PA, and a lack of access to healthful foods, all of which hinder residents from following ACS’s health behavior guidelines [28, 29]. ADI measures a neighborhood’s deprivation level with 17 socioeconomic indicators [30]. In the general population, residents of neighborhoods with high ADI (i.e., high deprivation level) have a high heavy drinking rate, physical inactivity, an increased obesity rate, and poor nutrition [31, 32]. PCa patients and their partners might be motivated by the PCa experiences to adopt healthful behaviors [33] but face challenges relevant to social and neighborhood barriers [34, 35]. However, it is unknown how ADI differentiates the PCa patients’ and partners’ health behaviors.

Black/African Americans (AA) have higher PCa incidence and mortality rates and are diagnosed at a younger age as compared with their White counterparts [36]. Aligned with these disparities, low adherence to ACS-recommended health behaviors has been observed among Black/AAs with PCa [16, 37, 38]. However, the identified racial differences in health behaviors may be partially attributed to ADI since Black/AAs often experience greater socioeconomic disadvantages than their White counterparts [39, 40]. Meanwhile, other cultural factors, such as food cultures [41], hair maintenance [42], and racism [43], may also affect Black/AAs’ health behaviors. Thus, this study aimed to examine the association of race (Black/AA vs. White) with the health behaviors of PCa patients and partners, controlling for their socioeconomic conditions (i.e., ADI and employment) and role (patient vs. partner caregiver). Understanding this association will help to inform culturally and socially appropriate behavioral interventions targeting PCa patients and partners, especially those self-identifying as Black/AAs, in future studies.

Overall, no study has focused on how multi-level factors (i.e., role, ADI, and race) simultaneously and independently affect the health behaviors and body mass index (BMI) of PCa patients and partners. Our study seeks to fill this gap by examining the associations of role (patient vs. partner), ADI (High ADI vs. Low ADI), and race (Black/AA vs. White) with health behaviors and BMI, controlling for age, comorbidity index, and employment status. The health behaviors include smoking, alcohol consumption, diet quality, sedentary behaviors, and PA. The control variables were selected due to their significant impact on these health behaviors (smoking [44, 45], alcohol consumption [46], diet [47], sedentary behaviors [48, 49], and PA [34, 50]).

## Methods

### Design and subjects

We analyzed the baseline data collected in a randomized controlled trial titled “Prostate Cancer Education & Resources for Couples (PERC)” (NR016990, PI: Song) [51]. For this randomized trial, we used the North Carolina Central Cancer Registry Rapid Case Ascertainment (RCA) to identify patients with localized PCa who were 40- to 75-year-old and had completed their curative PCa treatments within 16 weeks. We mailed a letter to their authorizing medical providers to obtain passive consent to contact these patients. If their medical providers returned no declination, we mailed a recruitment package to each patient to ask for their interest in participating in this study and also their permission to contact their intimate partner to participate in the study. Then we obtained consent from both patients and partners for study participation. We have reported the detailed recruitment strategies and eligibility criteria in the protocol of this study [51]. We achieved an enrollment rate of 78.4% (280 enrolled dyads out of 357 dyads whom we contacted).

### Data collection

We collected the baseline data immediately after dyads were enrolled in the study. We used a survey administered via telephone or online to collect the data based on dyads’ preferences. For the phone call survey, we asked PCa patients and partners the survey questions via telephone and entered the data into the secure REDCap database. For the online survey, we sent a unique REDCap survey link to PCa patients and partners for self-administration of the surveys. Approximately 77.4% and 22.4% of patients and partners completed the baseline surveys via phone and online, respectively, plus one using both methods to complete the surveys.

### Measurements

#### Role

We determined the role (PCa patient vs. partner) when we recruited dyads.

#### ADI

We used the 9-digit zip codes in patients’ and partners’ physical addresses to identify their ADI on the Neighborhood Atlas®-2019 ADI ranking in North Carolina [52], where 17 socioeconomic measures composed the ADI ranking [30]. The ADI ranking ranges from 1 to 10, with higher scores indicating a higher deprivation level. In this study, we used patients’ ADI to determine their partners’ ADI because 98% of the PCa patients and partners reported the same physical address. We categorized dyads with an NC state ADI ranking larger than 3 (median of the sample) into the high ADI group, while the remainder were the low ADI group [30].

#### Race

We asked dyads to self-report their race by selecting one from the options “White,” “Black/AA,” “American Indian or Alaska Native,” “Asian,” “Native Hawaiian or Other Pacific Islander,” and “other” in response to the question “What is your race?” in the demographic questionnaire. We only included the dyads self-identifying as “White” or “Black/AA” in the analysis as the size of other racial groups was < 10 in this sample.

#### Smoking

We assessed patients’ and partners’ smoking history using the yes/no question: “During your lifetime, have you smoked at least 100 cigarettes?” If they responded “yes” to this question, we asked additional questions to assess their smoking amount and duration. We assessed smoking amount with the question, “On average, how many cigarettes did you smoke per day during the entire time that you smoked?” answered by selecting one from four levels of cigarette numbers: “half pack,” “1 pack,” “2 packs,” and “2 or more packs” or “don’t know.” We asked patients and partners to self-report smoking duration in years. We also used a yes/no question, “Do you currently smoke?” to measure their current smoking status.

#### Alcohol consumption

We assessed alcohol consumption and frequency using the yes/no question “Do you ever drink alcohol?” and the question “How often in the past year have you failed to do what was expected of you because of alcohol?” answered by “not at all,” “occasionally,” or “frequently.” We also assessed their changes in alcohol consumption using the question, “To what extent has your alcohol use changed within the last 12 months?” answered by “decreased,” “increased,” or “about the same.”

#### Diet quality

We used adherence to the Mediterranean diet as a proxy for diet quality in this study, measured with the validated modified Mediterranean Diet Adherence Screener (MEDAS) [53]. This screener includes 12 questions about food consumption frequency and two questions about food intake characteristics of the Spanish Mediterranean diet. We scored each question with a “0,” “1,” or “don’t know.” The total score ranges from 0 to 14, with a higher value indicating a higher diet quality. The Cronbach’s alpha is 0.61 for patients and 0.63 for partners, calculated using the sample of this study.

#### Sedentary behaviors

We measured sedentary behaviors using the Measure of Older Adults’ Sedentary Time (MOST) scale [54, 55]. This scale assesses whether and how long individuals engage in six types of sedentary behaviors (watching TV/videos, using computer/Internet, reading, socializing with family/friends, transportation, and hobbies) in the last week, with one “other” option for them to report other types of sedentary behaviors. The test–retest reliability reported by previous studies is above 0.75 for watching TV, using computers, and reading [55]. We calculated the duration of each sedentary behavior by summing up the daily time and reported it as hours per week.

#### Physical activity

We assessed PA using the modified RESIDEntial Environment (RESIDE) questionnaire [56], a 16-item scale testing the length of time spent on PA in a usual week. The scale included four domains: walking for transportation, walking for recreation, moderate PA, and vigorous PA, with moderate or higher test–retest reliability for walking and moderate PA reported by previous studies [56]. This modified scale also measured the level of confidence to exercise more if patients or partners want to (“How confident are you that you could exercise more if you wanted to?”), answered by “very confident,” “somewhat confident,” “not at all confident,” “don’t know,” or “refused to answer.”

#### BMI

We calculated BMI with patients’ and partners’ self-reported current height and weight in the demographic questionnaire using the formula weight (kg)/[height (m)]^2^ [57].

#### Covariates

Covariates included age, employment status, and comorbidity. We calculated age with the self-reported date of birth in the demographic questionnaire. We assessed their employment status with a yes/no question, “Are you currently employed?” We measured comorbidity using the Charlson Comorbidity Index (CCI) [58]. We calculated CCI by summing up the comorbid conditions that individuals reported as “yes” [58]. The Cronbach’s alpha of CCI was 0.66 for patients and 0.71 for partners in this sample.

### Statistical analysis

We only included dyads in which patients or partners self-identified as “White” or “Black/AA” in the data analysis as the number of dyads of other races was much smaller than the two groups (*n* < 10). We used chi-square tests (for categorical variables) and *t*-tests (for numerical variables) to compare the demographic characteristics of PCa patients and partners grouped by ADI or race. For all outcome variables, the missing date rate is below 5%.

Considering the nested structure of prostate cancer (PCa) patients and their partners of varying races within neighborhoods of different deprivation levels [59], we utilized multi-level models (MLMs) to investigate the impact of role, ADI, and race on health behavior outcomes. We incorporated age, employment status, and CCI [55] as covariates into the analysis.

Our final model was reached through an iterative process, initially including the main effects and interactions of any two of the three predictor variables: role, ADI, and race (i.e., two-way interactions). We kept the interaction model if any two-way interactions were significant. If no two-way interactions were significant, we removed the interaction terms to achieve the final main effect models.

We treated categorical and continuous outcome variables separately. For continuous outcome variables, we calculated least-square means and estimated mean differences between groups (patient vs. partner, high vs. low ADI, Black/AA vs. White). For categorical outcome variables, we computed the adjusted odds ratio (OR) between the aforementioned groups. We used partner, low ADI, and White as the reference level for each group, respectively.

Among categorical outcome variables, we used “no” response as the reference for the variables “smoked >  = 100 cigarettes,” “smoking now,” “ever drink alcohol,” sedentary behaviors, PA, and “confident to exercise more.” For packs of cigarettes smoked per day, the lowest level (“half pack”) was the reference level. The reference levels for frequency of alcohol-induced duty lapses in the past year and changes in alcohol intake over the last 12 months were “not at all” and “decreased,” respectively.

We performed all tests and analyses using SAS (version 9.4, SAS Institute, Inc., Cary, NC) with a two-sided significance level set at an alpha of 0.05.

## Results

### Sample characteristics

Among 254 couples included in this analysis, 202 PCa patients and 205 partners self-identified as White, and 52 PCa patients and 49 partners self-identified as Black/AA. A total of 98.8% of the couples were racially concordant (202 White and 49 Black/AA couples). One hundred and ten couples had high ADI, and 144 couples had low ADI. All patients were male, and 99.61% of the partners were female. As outlined in Table 1, significant differences in education level and family income were found between high and low ADI groups (*p*s < 0.05). Significant differences in education level and family income were also observed between White and Black/AA groups except that the differences in education level between White partners and Black/AA partners were not significant. The CCI of the dyads living in the high ADI areas was significantly higher than that of those living in the low ADI areas (*p* < 0.05).

**Table 1:** Sample characteristics grouped by role, area deprivation index (ADI), and race

	Patients										Partners									
	White		Black/AA			High ADI		Low ADI			White		Black/AA			High ADI		Low ADI		
Characteristics	*N*	*%*	*N*	*%*	*p*	*N*	*%*	*N*	*%*	*p*	*N*	*%*	*N*	*%*	*p*	*N*	*%*	*N*	*%*	*p*
Gender																				
Male	202	79.5	52	20.5	NA	110	43.3	144	56.7	NA	1	100	0	0	1	0	0	1	100	1
Female	0	0	0	0		0	0	0	0		204	80.6	49	19.4		110	43.5	143	56.5	
Education																				
Less than college	69	69.7	30	30.3	0.01*	65	65.7	34	34.3	< .0001*	81	77.9	23	22.1	0.72	53	51	51	49	0.03*
Bachelor’s	65	85.5	11	14.5		12	15.8	64	84.2		58	80.6	14	19.4		23	31.9	49	68.1	
Graduate	56	88.9	7	11.1		23	36.5	40	63.5		47	83.9	9	16.1		21	37.5	35	62.5	
Other	11	73.3	4	26.7		10	66.7	5	33.3		19	86.4	3	13.6		13	59.1	9	40.9	
Family income																				
< = $90,000	87	73.1	32	26.9	0.03*	70	58.8	49	41.2	< .0001*	81	73	30	27	0.03*	62	55.9	49	44.1	< .0001*
> $90,000	104	86.7	16	13.3		34	28.3	86	71.7		102	86.4	16	13.6		36	30.5	82	69.5	
Type of treatment																				
Surgery	149	78.8	40	21.2	0.72	76	40.2	113	59.8	0.09	NA		NA		NA	NA		NA		NA
Radiation	53	81.5	12	18.5		34	52.3	31	47.7											
Characteristics	*M*	*SD*	*M*	*SD*	*p*	*M*	*SD*	*M*	*SD*	*p*	*M*	*SD*	*M*	*SD*	*p*	*M*	*SD*	*M*	*SD*	*p*
Age	64.4	6.5	63.6	6.7	0.6	64.1	6.8	64	6.3	0.85	61.7	7.3	60.1	7	0.19	61.1	7	61.6	7.5	0.54
CCI	4.3	1.9	4.3	1.9	0.98	4.6	2	4	1.8	0.01*	3.8	1.9	4.1	2.4	0.38	4.2	2	3.6	2	0.02*
Relationship length (years)	34.3	12.4	30.5	12.2	0.05	32.8	12.4	34.1	12.4	0.4	34.5	12.4	31.4	12.1	0.11	33.3	11.9	34.4	12.6	0.48
Household size	3.4	0.9	2.5	1.2	0.27	2.5	1.1	2.4	0.8	0.68	2.5	1.1	2.5	0.8	0.89	2.5	1.2	2.5	0.9	0.7

*N* number, *M* mean, *SD* standard deviation, % percentage, *Black/AA* Black/African American, *ADI* area deprivation index, *CCI* Charlson Comorbidity Index; *p <.05

### Health behaviors and BMI

**Fig. 1:**
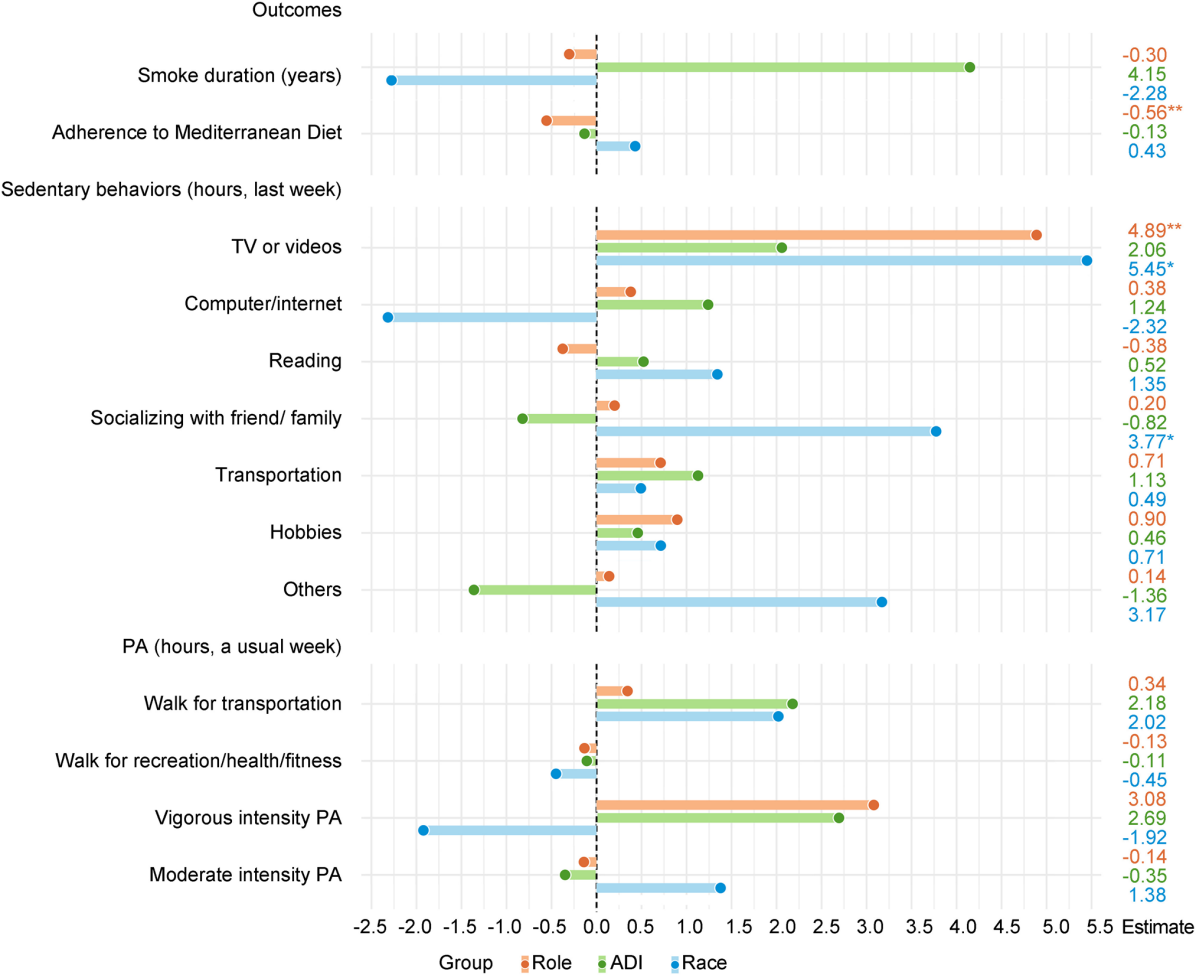
The estimated mean differences in continuous health behavior outcome variables by role, area deprivation index (ADI), and race as estimated by main effect models. *p < .05, **p < .01, ***p < .001; PA physical activity; partner, low ADI, and White were used as reference for role, ADI, and race, respectively; smoke duration, time spent on sedentary behaviors, and hours of physical activity were only collected among dyads who reported engagement in these behaviors

**Fig. 2:**
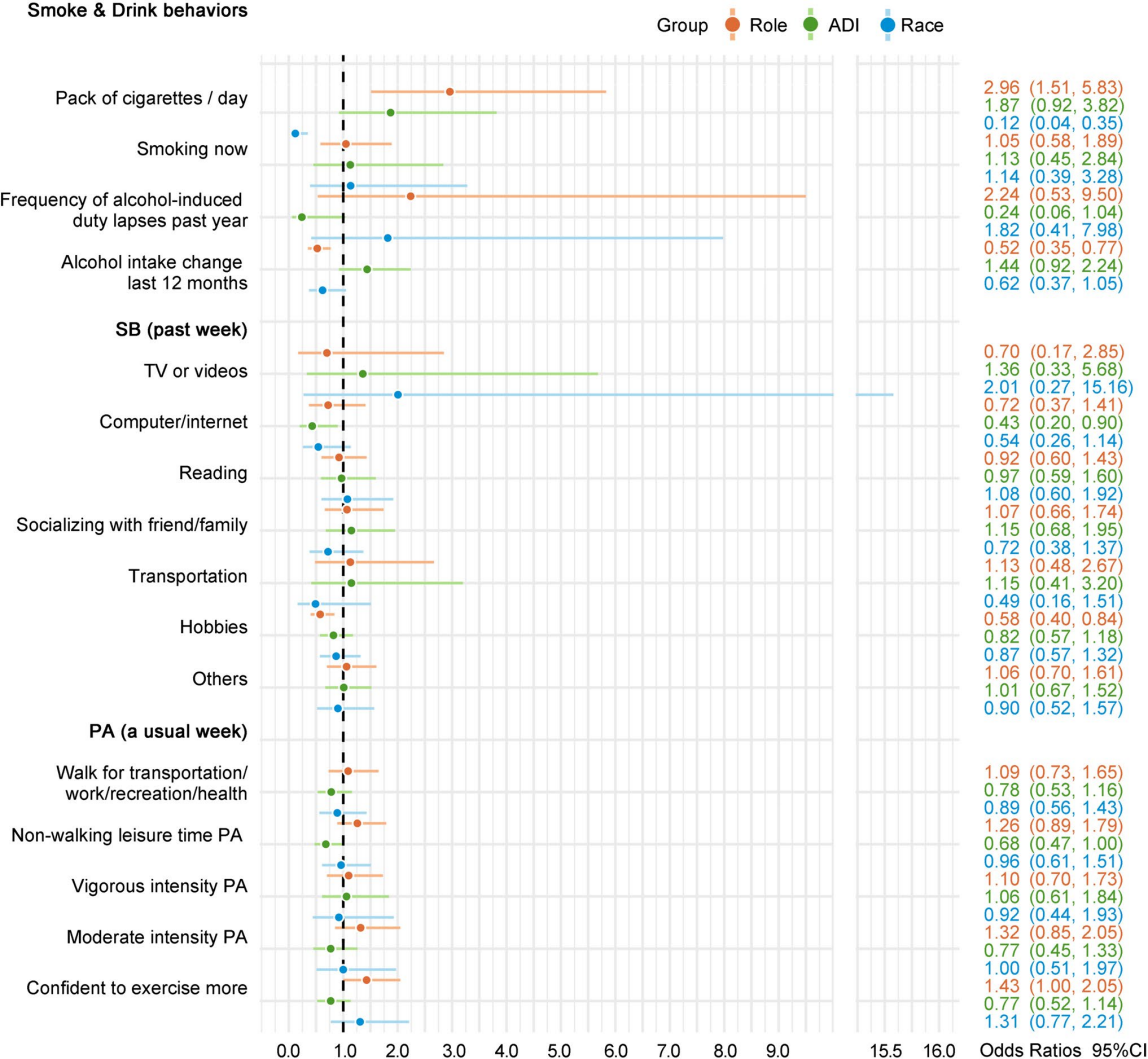
Adjusted odds ratio of categorical health behavior outcome variables associated with role, area deprivation index (ADI), and race as estimated by main effect models. PA physical activity; SB sedentary behaviors; CI confidence interval; partner, low ADI and White were reference for role, ADI, and race, respectively; “no” level was used as the reference for smoking, sedentary behaviors, and physical activity, whereas the lowest level was used as the reference for packs of cigarettes smoked per day (“half pack”), frequency of alcohol-induced duty lapses in the past year (“not at all”), alcohol intake change in the last 12 months (“decreased”), and confident to exercise more (“not at all”); a dot represents adjusted odds ratio (OR) and the straight line that cross the dot represents 95% CI of the adjusted OR. If the 95% CI includes 1 (e.g., 95% CI 0.9–1.1), this indicates the adjusted OR is not statistically significant

The final models for all outcome variables were main effect models except for “smoked >  = 100 cigarettes,” “ever drink alcohol,” and BMI because significant two-way interaction effects were only detected for the three outcome variables (Table 2, Figs. 1 and 2).

**Table 2:** Role, area deprivation index (ADI), and race effects on smoking, alcohol consumption, and body mass index (BMI) as estimated by multi-level models with interaction effects

**Outcome**	**Role**		**ADI**		**Race**		Role*race		
	**Adjusted *OR***	**95%*CI***	**Adjusted *OR***	**95%*CI***	**Adjusted *OR***	**95%*CI***	**Estimated odds**		**95% *CI***
**Smoked > = 100 cigarettes**	1.12	(0.78, 1.60)	1.18	(0.77, 1.82)	0.51	(0.24, 1.11)	White patient	4.03	(1.46, 8.88)
							Black/AA patient	7.47	
							White partner	46.91	
							Black/AA partner	87.12	
	**Role**		**ADI**		**Race**		ADI*race		
	**Adjusted *OR***	**95%*CI***	**Adjusted *OR***	**95%*CI***	**Adjusted *OR***	**95%*CI***	**Estimated odds**		**95% *CI***
**Ever drink alcohol**	1.34	(0.94, 1.91)	0.25	(0.14, 0.48)	0.25	(0.11, 0.59)	White dyad in high ADI	0.91	(1.17, 11.01)
							Black/AA dyad in high ADI	0.83	
							White dyad in low ADI	46.01	
							Black/AA dyad in low ADI	42.02	
	**Role**		**ADI**		**Race**		Role*ADI		
	**Estimated mean differences**	***p***	**Estimated mean differences**	***p***	**Estimated mean differences**	***p***	**Estimated means**		***p***
**BMI**	1.84	< 0.01	2.19	< 0.01	2.98	< 0.01	Patient in high ADI	29.56	0.04
							Partner in high ADI	30.89	
							Patient in low ADI	28.66	
							Partner in low ADI	28.17	

*ADI* area deprivation index, *OR* odds ratio, *CI* confidence interval, Black/AA Black/African Americans, *BMI* body mass index; partner, low ADI, and White were used as reference for role, ADI, and race, respectively; “no” level was used as the reference for “smoked >  = 100 cigarettes” and “ever drinking alcohol”; if the 95% CI includes 1 (e.g., 95% CI 0.9–1.1), this indicates the adjusted OR is not statistically significant

#### Smoking

An interaction model was used to estimate role, ADI, and race effects on “smoked >  = 100 cigarettes” because significant role-race interaction effects were noted for this outcome variable (Table 2). As estimated by this interaction model, partners who self-identified as Black/AAs showed the highest odds of having smoked >  = 100 cigarettes (87.12), followed by White partners (46.91), Black/AA PCa patients (7.47), and White patients (4.03). No significant main effects of role, ADI, or race were found by this model (Table 2).

Main effect models examined the role, ADI, and race effects on the remaining smoking behaviors (i.e., packs of cigarettes smoked per day, smoking now, and smoking duration) due to no significant interaction effects detected. Patients smoked significantly more packs of cigarettes per day than their partners (adjusted OR 2.96, 95% CI [1.51, 5.83]; Fig. 2). Black PCa patients and partners smoked significantly fewer packs of cigarettes than their White counterparts (adjusted OR 0.12, 95% CI [0.04, 0.35]; Fig. 2). No significant effects were observed for current smoking status or duration (Figs. 1 and 2).

#### Alcohol consumption

As indicated in Table 2, significant ADI-race interaction effects were identified on “ever drink alcohol” (95% CI, 1.17–11.01; Table 2). As estimated by this interaction model, patients and partners who self-identified as White and living in low ADI areas had the highest odds of ever drinking alcohol (46.01). Subsequently, those self-identifying as Black/AA and living in low ADI areas had the second-highest odds (42.02), followed by White dyads who lived in high ADI areas (0.91) and Black/AA dyads living in high ADI areas (0.83). This interaction model also detected significant ADI and race main effects on ever drinking alcohol. PCa patients and partners who lived in high ADI neighborhoods had significantly lower odds of ever drinking alcohol in comparison to those living in low ADI neighborhoods while controlling for their role, race, and the ADI-race interactions (adjusted OR 0.25, 95% CI [0.14, 0.48], Table 2). Black/AA dyads were less likely to drink alcohol than their White counterparts independent of role, ADI, and ADI-race interaction effects (adjusted OR 0.25, 95% CI [0.11, 0.59], Table 2).

Additionally, as estimated by a main effect model, patients had significantly lower odds of increasing or maintaining alcohol intake than their partners in the last 12 months (adjusted OR 0.52, 95% CI [0.35, 0.77]; Fig. 2). No significant interaction or main effects were found on the frequency of alcohol-induced duty lapses in the last year.

#### Diet quality

PCa patients’ Mediterranean diet score was 0.56 lower than their partners (*p* < 0.01) as estimated by a main effect model, indicating that partners had higher diet quality than PCa patients. No significant interaction, ADI, or race effects were detected on diet quality (Fig. 1).

#### Sedentary behaviors

Main effect models were used to estimate role, ADI, and race effects on sedentary behaviors in the past week because no significant interaction effects were detected (Figs. 1 and 2). PCa patients reported a significantly lower likelihood of doing sedentary hobbies than their partners (adjusted OR 0.58, 95% CI [0.40, 0.84]; Fig. 2). Compared with dyads living in low ADI neighborhoods, dyads living in high ADI areas were less likely to use computers/Internet (adjusted OR 0.43, 95% CI [0.20, 0.90]; Fig. 2). Among those who reported doing sedentary behaviors, PCa patients spent 4.89 h more watching TV/videos than their partners (*p* < 0.01; Fig. 1). Black/AA dyads spent 5.45 and 3.77 more sedentary hours watching TV/videos and socializing with friends/family than their White counterparts, respectively (*ps* < 0.05; Fig. 1).

#### Physical activity

No significant interaction effects were found on any PA behaviors. Main effect models showed significant role and ADI effects on PA. Compared with partners, PCa patients reported significantly higher confidence to exercise more (adjusted OR 1.43, 95% CI [1.00, 2.05]; Fig. 2). The odds of doing other leisure time PA excluding walking among the high-ADI group were significantly lower than the low-ADI group (adjusted OR 0.68, 95% CI [0.47, 1.00]; Fig. 2).

#### BMI

As estimated by an interaction model, significant role-ADI interaction effects and the role, ADI, and race main effects were detected on BMI. For role-ADI interaction, partners who lived in high ADI areas reported the highest BMI, with high ADI patients reporting the second highest BMI; the partners who lived in low ADI areas had the lowest BMI (*p* = 0.04, estimated BMI mean scores of high ADI partners 30.89; high ADI patients 29.56; low ADI patients 28.66; low ADI partners 28.17; Table 2). Regarding main effects, patient, high ADI, and Black/AA were independently associated with a significantly higher BMI by 1.84 kg/m^2^, 2.19 kg/m^2^, and 2.98 kg/m^2^, respectively, compared to partners, low ADI, and White (*p*s < 0.01; Table 2).

## Discussion

In this study, we used MLMs to examine the effects of socioeconomic deprivation at the neighborhood level and role/race effects at the individual level on the ACS-recommended health behaviors and BMI among PCa patient-partner dyads recruited in North Carolina, USA. We detected significant role*race interaction effects on smoking, ADI*race effects on alcohol consumption, and role*ADI effects on BMI among PCa patients and partners, in which Black/AA partners reported the highest odds of smoking >  = 100 cigarettes, White dyads living in low ADI neighborhoods had the highest odds of ever drinking alcohol, and partners with high ADI reported the highest BMI. We also identified significant differences in smoking amount, alcohol consumption changes, diet quality, sedentary behaviors, and confidence to do PA between PCa patients and partners. We found significant independent associations of high ADI with lower odds of ever drinking alcohol, using computers/Internet, and doing non-walking leisure-time PA in comparison to low ADI after controlling for their role and race; notably, these behaviors are all resource-dependent as they are relevant to alcohol availability, accessibility to computers/Internet, and resources for PA. Furthermore, we observed significantly lower engagement in smoking and alcohol consumption and more sedentary time among dyads who self-identified as Black/AA than their White counterparts after controlling for their role and ADI. Altogether, these findings indicate that role, ADI, and race are independently and interdependently associated with health behaviors and BMI of PCa patients and their partners. Effective behavioral interventions should address challenges relevant to neighborhood resources and be culturally tailored to the role- and race-related needs of PCa patients and partners.

Compared to previous studies, the dyads in this clinical trial reported healthier lifestyle behaviors (Supplementary Table 1 presents the description of each health behavior grouped by role, ADI, and race). In our study, there was a lower proportion of current smokers (6.4% vs. 8.8%) and a higher proportion of doing moderate-vigorous PA for >  = 150 min per week (66.5% vs. 41.2%) among PCa patients than cancer patients in previous studies [44, 60]. There was also a lower proportion of current smokers (6.7% vs. 11.2%) and a higher proportion of doing >  = 150-min moderate-vigorous PA weekly (59.4% vs. 50.5%) among the partners of this sample than the general female population [61, 62]. Furthermore, both PCa patients and partners adhered to the Mediterranean diet to a greater extent than the general population (8.16 vs. 6.22, 8.55 vs. 6.22) [63]. These findings might suggest that the patients who recently completed PCa treatment and their partners might have adopted healthier lifestyle behaviors as compared to the general population, as the cancer diagnosis and treatment created a series of “teachable moments” for patients and their families [33]; they may receive preventive health care services and education from healthcare professionals while managing their care transitions and comorbid conditions [64, 65]. Nevertheless, we must carefully interpret these comparison results concerning the limitation of descriptive versus inferential statistics, the potential for recall bias, and the social desirability of the self-report instruments.

This study found significantly different health behaviors between PCa patients and partners. Partners had significantly healthier smoking habits and dietary behaviors; however, patients were less likely to increase or maintain alcohol consumption and more confident in doing PA. Considering that all partners but one were female, and all patients were male, some differences in health behaviors between patients and partners in this study might be aligned with the gender differences in health behaviors. For example, females usually smoke fewer cigarettes [66], have a greater awareness of healthy eating [67], and report less confidence in doing PA [68] than males. However, in addition to the gender effects, their role as a patient or partner could also contribute to the differences in their health behaviors due to the different challenges patients or partners face. For example, partners do more sedentary hobbies possibly to cope with the increased mental distress, as studies have shown that partners suffer from higher mental distress than patients [59, 69]; however, doing these sedentary hobbies also increases their likelihood of having a sedentary lifestyle. Patients may be motivated by their PCa diagnosis to decrease alcohol consumption, while partners may not spare an effort to maintain their own health as they cope with the patient’s PCa and respond to increased caregiver burden and family commitments [70, 71]. In summary, concerning the gender-specific nature of PCa, gender-related factors (e.g., gender norms [72]) and role-differentiated needs should be considered to promote healthful behaviors among PCa patients and partners. Additional research is needed to identify the separate sex and role effects on health behaviors to develop effective couple-based health behavior interventions for PCa patients and partners.

This study found PCa patients and partners living in high ADI areas had a significantly lower likelihood of ever drinking alcohol, using computer/Internet, and doing non-walking leisure-time PA, all of which are resource-dependent. That is to say, the engagement of PCa survivors and partners in these behaviors is affected by their accessibility to social and neighborhood resources, which varies across geographical areas. For example, the less use of computers and Internet in high ADI neighborhoods may be related to the limited accessibility to the network infrastructure. This further hinders PCa patients and partners living in high ADI areas from accessing online social support [73, 74], telehealth services [75], and Internet-based health promotion information or programs [76], when they have limited social support and resources within their neighborhoods [77]. The lack of social interactions and support in high ADI neighborhoods is also confirmed by the significant relationship of high ADI to lower odds of ever drinking alcohol after controlling for race and role in our study, which is consistent with the results among the general population [78] since alcohol consumption usually occurs in social context [78]. Previous studies have also observed a higher likelihood of heavy drinking in the general population living in high ADI neighborhoods [78], which is not observed in our study; possibly due to the PCa experiences motivating PCa patients and partners to follow healthy drinking behaviors. In addition, a highly deprived neighborhood may have an unsafe environment or lack PA facilities, such as parks, playgrounds, and gyms [77], which decreases residents’ probability of doing PA. Overall, the deficient social and environmental resources in high ADI areas may be directly related to adverse health outcomes [59, 79] or via altering one’s awareness, emotional states, and ability to adopt and maintain healthy behaviors[80]. Still, for future studies, it is essential to specify the indicators within a neighborhood that affect its residents’ health behaviors to design multi-level behavioral intervention studies that target these social and neighborhood barriers.

After controlling for role and ADI, this study still observed racial differences in health behaviors similar to previous studies, in which Black/AA patients and partners reported lower smoking amount, a lower likelihood of drinking alcohol, and more time spent on sedentary behaviors than their White counterparts [37, 81–84]. Our study controlled for the effects of socioeconomic conditions (i.e., ADI), while previous studies did not, thus indicating that the racial differences in health behaviors were not solely attributed to socioeconomic causes. For example, one factor that may relate to the more sedentary lifestyle among Black/AA patients and partners is the lack of communication regarding PA and sedentary behaviors with their physicians [85]. The higher probability of drinking alcohol among Whites than Black/AAs after controlling for socioeconomic disadvantages, consistent with findings among the general population [86], may be partially related to the fact that White men were more likely to link masculinity to drinking alcohol than Black/AA men [87]. As diverse engagement in health behaviors contributes to inequitable health outcomes across racial groups [36, 37, 84, 88], future intervention studies should use culturally adapted strategies to promote a healthy lifestyle and achieve health equity across racial groups.

Although ACS has combined sedentary behaviors and PA as “being physically active” in their recommendations for cancer prevention and survivorship [3], sedentary behaviors can lead to declined physical functioning, weight gain, and cardiovascular diseases exclusive of PA [89]. Our study found that PCa patients and their partners had a highly sedentary lifestyle with considerable weekly hours spent on sedentary behaviors (Supplementary Table 1). Considering the lack of knowledge about sedentary behaviors and the confusion between sedentary behaviors and PA that generally existed in this population [90], more studies should focus on minimizing sedentary lifestyles. Further, in this study, not all role, racial, and ADI effects on sedentary behaviors were consistent with those on PA. For example, Black/AA dyads spent significantly longer sedentary time than their White counterparts, with no reported significant differences in the time spent on any PA between the two racial groups after controlling for role and ADI. These findings revealed that PA and sedentary behaviors were affected by different factors across racial groups. For example, Black/AA individuals may participate in regular PA and have a sedentary lifestyle preferring individual activities (e.g., watching TV/videos) or non-threatening social environments (e.g., socializing with family/friends) at the same time [91]. Considering the lower efficacy of existing interventions in reducing sedentary behaviors than promoting PA [92], future studies should clarify the independent factors influencing sedentary behaviors across racial groups to develop racially and culturally appropriate behavioral interventions to minimize racial differences in physical inactivity among PCa patients and partners.

The results from our study corroborate those from previous research that Black/AA and high ADI were associated with higher BMI in comparison to White race and low ADI, respectively [37, 83, 93]. Also consistent with the findings from Byrd et al.’s work (2017), we found that the sample mean BMI of Black/AA dyads was within the obese range (Supplementary Table 1) [83]. Given the adverse effect of overweight/obesity on general health and PCa survivorship [94], the higher BMI in the Black/AA or high ADI group may contribute to cancer and health disparities in this population [79, 95, 96]. For example, weight gain mediates the relationship between a sedentary lifestyle and increased aggressiveness of PCa among Black/AA PCa patients [97]; thus, maintaining a healthy weight, as recommended by ACS, will help mitigate adverse outcomes during PCa survivorship [94] and a healthy weight can be achieved by engaging in healthful behaviors [12]. Besides health behaviors, stress has also been identified as a significant risk factor for obesity [98], which may explain the higher BMI observed among partners living in high ADI neighborhoods than other groups in our study. To note, in this sample, the partners living in high ADI areas were all female. Research has found that they usually suffer from a higher stress level than PCa patients [59, 69]. Those living in high ADI areas have limited access to supportive resources [99, 100], which worsens their caregiving burden and mental distress and increases their susceptibility to obesity and weight gain. Overall, multi-level intersecting factors, such as neighborhood resources, mental distress, and health behaviors, ought to be addressed to facilitate weight control for PCa patients and partners to benefit their health and mitigate PCa and health disparities. Nonetheless, the BMI differences between Black/AA and White individuals may not represent the differences in adiposity between the two racial groups. Black/AAs have a lower percentage of body fat, greater bone mineral density and body protein context, and different body shapes compared to their White counterparts of the same BMI [101, 102]. Future studies should consider multiple measures of adiposity, such as waist circumference and mean lean mass, to increase the accuracy of identifying racial disparities in obesity among PCa patients and partners [103].

### Limitations

A few limitations must be taken into consideration when interpreting these results. First, data from approximately 33.6% of the dyads (*n* = 94) were collected during the COVID-19 pandemic [104]. Social distancing and sheltering in place may have affected health behaviors, such as increasing home cooking and sedentary behaviors and disrupting PA routines and access (e.g., gym, parks) [105]. Alcohol consumption also increased with the experience of psychological distress during the pandemic [105]. Additionally, there was a notable increase in yearly BMI gain during the COVID-19 pandemic compared to the previous year [106]. Considering that Black/AA individuals and residents in high-ADI neighborhoods experienced increased mental distress during the pandemic [107, 108], the COVID-19 pandemic may have partially contributed to the identified ADI and race effects in this study. Second, it is difficult to disentangle the role effects from gender because the patients were 100% male, and the partners were 99.6% female. Third, although we collected the health behavior data among post-treatment PCa patients and their partners, they were asked to recall their current behaviors (e.g., right now or in the past week), general behaviors (e.g., in a usual week or their lifetime ever), or in the past year or 12 months. In other words, these measured health behaviors may occur before the completion of PCa treatments. As health behaviors may differ by treatment type, future research should investigate the effect of various treatments on health behaviors. Finally, measurement errors existed as patients and partners completed the baseline surveys via telephone or an online platform, by which their responses may differ. Further, these surveys were all self-reported with a risk of recall bias and social desirability in this study. Yet, all scales had good reliability and validity as calculated using the sample of this study or by previous studies.

### Strengths

Nonetheless, this study has multiple strengths that advance the research in health behavior during cancer survivorship. This study included all the health behaviors related to survival outcomes and quality of life as identified in ACS’s guidelines for cancer prevention and survivorship [3]. Together with PCa patients, we recruited their partners as patient-partner dyads. Given the nested structure of the data, the multi-level models strengthened this study, as this method accounted for both the variability within each dyad and the variability between dyads. The racial distribution of this sample well represents the local population, as the proportion of Black/AAs in the sample (20%) aligns with that in North Carolina.

## Conclusion

This study identified significant role*race interaction effects on smoking, ADI*race effects on alcohol consumption, and role*ADI effects on BMI. This study also revealed significant main effects of role, ADI, or race on smoking, alcohol consumption, diet quality, sedentary behaviors, or PA among PCa patients and their partners.

These findings inform the development of multi-level, culturally adapted, and tailored interventions to promote ACS-recommended health behaviors among PCa patients and their partners and achieve equitable improvements in the quality of survivorship across racial groups and neighborhoods. Future behavioral interventions should address individual (patient or partner) needs, control the barriers related to social and neighborhood resources, and consider racially aligned factors and challenges that affect these health behaviors. Our findings also addressed a need for interventions specifically targeting sedentary behaviors apart from PA and validated objective measurements for health behaviors and adiposity beyond the self-report surveys and BMI, respectively.

## Supplementary Information

Below is the link to the electronic supplementary material.

Supplementary file1 (DOCX 48 KB)

## Data Availability

The dataset that is analyzed in this work is available from the corresponding author upon reasonable request.
