# Supplementary material for: Associations of role, area deprivation index, and race with health behaviors and body mass index among localized prostate cancer patients and their partners
Source: J Cancer Surviv. 2024 Jun 18;20(1):300–14. doi: 10.1007/s11764-024-01625-z (PMC12906562; doi:10.1007/s11764-024-01625-z)
Supplement: Supplementary file 1 — Supplementary file1 (DOCX 48 KB) [file 11764_2024_1625_MOESM1_ESM.docx]

Article title: Associations of Role, Area Deprivation Index, and Race with Health Behaviors and Body Mass Index among Localized Prostate Cancer Patients and Their Partners

Journal name: Journal of Cancer Survivorship

Authors:

Jingle Xu, MSN, MHS

Chunxuan Ma, MS

Rachel Hirschey, PhD, RN, FAAN

Jia Liu, PhD, MSN, RN

Daria B Neidre, PhD, MA

Matthew E. Nielsen, MD

Thomas C. Keyserling, MD, MPH

Xianming Tan, PhD

Lixin Song, PhD, RN, FAAN

Corresponding author:

Lixin Song

School of Nursing, The University of Texas Health Science Center at San Antonio, San Antonio, TX

Email: songl2@uthscsa.edu

Supplemental Table 1. Prostate cancer patients and partners characteristics by role, area deprivation index (ADI), and race.

| Health Behaviors | | | Role | | | | Area deprivation index | | | | Race | | | |
| --- | --- | --- | --- | --- | --- | --- | --- | --- | --- | --- | --- | --- | --- | --- |
|  |  |  | Patient | | Partner | | High | | Low | | Black | | White | |
|  |  |  | *N* | *%* | *N* | *%* | *N* | *%* | *N* | *%* | *N* | *%* | *N* | *%* |
| Smoked >= 100 cigarettes | | |  |  |  |  |  |  |  |  |  |  |  |  |
|  | Yes | | 104 | 41.3 | 82 | 32.4 | 89 | 40.6 | 97 | 33.9 | 39 | 39 | 147 | 36.3 |
|  | No | | 148 | 58.7 | 171 | 67.6 | 130 | 59.4 | 189 | 66.1 | 61 | 61 | 258 | 63.7 |
| Packs of cigarettes smoked^§^ | | |  |  |  |  |  |  |  |  |  |  |  |  |
|  | < half pack | | 40 | 39.2 | 41 | 57.8 | 35 | 43.2 | 46 | 50 | 27 | 73 | 54 | 39.7 |
|  | 1 pack | | 45 | 44.1 | 27 | 38 | 31 | 38.3 | 41 | 44.6 | 9 | 24.3 | 63 | 46.3 |
|  | 2 packs | | 12 | 11.8 | 3 | 4.2 | 11 | 13.6 | 4 | 4.4 | 0 | 0 | 15 | 11 |
|  | >= 2 packs | | 3 | 2.9 | 0 | 0 | 3 | 3.7 | 0 | 0 | 0 | 0 | 3 | 2.2 |
| Smoking now | | |  |  |  |  |  |  |  |  |  |  |  |  |
|  | Yes | | 16 | 6.4 | 17 | 6.7 | 17 | 7.9 | 16 | 5.6 | 8 | 8 | 25 | 6.2 |
|  | No | | 233 | 93.6 | 237 | 93.3 | 199 | 92.1 | 271 | 94.4 | 92 | 92 | 378 | 93.8 |
| Ever drink alcohol | | |  |  |  |  |  |  |  |  |  |  |  |  |
|  | Yes | | 195 | 77.4 | 184 | 72.7 | 138 | 63.3 | 241 | 84 | 64 | 64 | 315 | 77.8 |
|  | No | | 57 | 22.6 | 69 | 27.3 | 80 | 36.7 | 46 | 16 | 36 | 36 | 90 | 22.2 |
| Frequency of alcohol-induced duty lapses | | |  |  |  |  |  |  |  |  |  |  |  |  |
|  | Not at all | | 186 | 95.9 | 138 | 97.9 | 124 | 98.4 | 200 | 95.7 | 57 | 95 | 267 | 97.1 |
|  | Occasionally | | 8 | 4.1 | 2 | 1.4 | 2 | 1.6 | 8 | 3.8 | 3 | 5 | 7 | 2.5 |
|  | Frequently | | 0 | 0 | 1 | 0.7 | 0 | 0 | 1 | 0.5 | 0 | 0 | 1 | 0.4 |
| Alcohol intake change last 12 months | | |  |  |  |  |  |  |  |  |  |  |  |  |
|  | Decreased | | 75 | 30.5 | 48 | 19.6 | 48 | 22.5 | 75 | 27 | 31 | 33 | 92 | 23.2 |
|  | About the same | | 166 | 67.5 | 189 | 77.1 | 158 | 74.2 | 197 | 71 | 61 | 64.9 | 194 | 74.1 |
|  | Increased | | 5 | 2 | 8 | 3.3 | 7 | 3.3 | 6 | 2.2 | 2 | 2.1 | 11 | 2.8 |
|  | | | *M* | *SD* | *M* | *SD* | *M* | *SD* | *M* | *SD* | *M* | *SD* | *M* | *SD* |
| Smoke duration (years)^§^ | | | 19.9 | 14.4 | 19.4 | 13.6 | 22.8 | 15.7 | 17 | 12 | 19.4 | 13.9 | 19.8 | 14.2 |
| Body mass index | | | 28.8 | 4.8 | 28.4 | 6.3 | 29.8 | 6.4 | 27.6 | 4.7 | 30.6 | 6.9 | 28.1 | 5.2 |
| Sedentary Behaviors (past week) | | | *N* | *%* | *N* | *%* | *N* | *%* | *N* | *%* | *N* | *%* | *N* | *%* |
|  | Watching TV/videos | |  |  |  |  |  |  |  |  |  |  |  |  |
|  |  | Yes | 249 | 98 | 249 | 98.4 | 217 | 98.6 | 281 | 97.9 | 100 | 99 | 398 | 98 |
|  |  | No | 5 | 2 | 4 | 1.6 | 3 | 1.4 | 6 | 2.1 | 1 | 1 | 8 | 2 |
|  | Using computer/Internet | |  |  |  |  |  |  |  |  |  |  |  |  |
|  |  | Yes | 226 | 90 | 235 | 93.6 | 192 | 87.7 | 269 | 95 | 88 | 87.1 | 373 | 93 |
|  |  | No | 25 | 10 | 16 | 6.4 | 27 | 12.3 | 14 | 5 | 13 | 12.9 | 28 | 7 |
|  | Reading | |  |  |  |  |  |  |  |  |  |  |  |  |
|  |  | Yes | 207 | 81.8 | 200 | 79.7 | 175 | 79.9 | 232 | 81.4 | 80 | 80.8 | 327 | 80.7 |
|  |  | No | 46 | 18.2 | 51 | 20.3 | 44 | 20.1 | 53 | 18.6 | 19 | 19.2 | 78 | 19.3 |
|  | Socializing with family/friends | |  |  |  |  |  |  |  |  |  |  |  |  |
|  |  | Yes | 219 | 86.2 | 217 | 86.1 | 190 | 86.4 | 246 | 86 | 83 | 83 | 353 | 87 |
|  |  | No | 35 | 13.8 | 35 | 13.9 | 30 | 13.6 | 40 | 14 | 17 | 17 | 53 | 13 |
|  | Transportation | |  |  |  |  |  |  |  |  |  |  |  |  |
|  |  | Yes | 243 | 96.4 | 242 | 96 | 211 | 96.3 | 274 | 96.1 | 94 | 94 | 391 | 96.8 |
|  |  | No | 9 | 3.6 | 10 | 4 | 8 | 3.7 | 11 | 3.9 | 6 | 6 |  | 3.2 |
|  | Hobbies | |  |  |  |  |  |  |  |  |  |  |  |  |
|  |  | Yes | 117 | 46.4 | 150 | 59.3 | 112 | 50.9 | 155 | 54.4 | 50 | 49.5 | 217 | 53.7 |
|  |  | No | 135 | 53.6 | 103 | 40.7 | 108 | 49.1 | 130 | 45.6 | 51 | 50.5 | 187 | 46.3 |
|  | Others | |  |  |  |  |  |  |  |  |  |  |  |  |
|  |  | Yes | 65 | 25.8 | 62 | 24.7 | 55 | 25.2 | 72 | 25.3 | 24 | 23.8 | 103 | 25.3 |
|  |  | No | 187 | 74.2 | 189 | 75.3 | 163 | 74.8 | 213 | 74.7 | 77 | 76.2 | 299 | 74.7 |
|  | Duration (hours, past week)^§^ | | *M* | *SD* | *M* | *SD* | *M* | *SD* | *M* | *SD* | *M* | *SD* | *M* | *SD* |
|  |  | Watching TV/videos | 22.5 | 19.6 | 18.2 | 13.2 | 22.5 | 20 | 18.7 | 13.7 | 25.4 | 26.5 | 19.1 | 13.2 |
|  |  | Using computer/Internet | 19.6 | 16 | 20 | 16.9 | 20.3 | 16 | 19.4 | 16.8 | 18.6 | 16.2 | 20.1 | 16.6 |
|  |  | Reading | 7.7 | 6.8 | 7.9 | 7.5 | 8.3 | 7.3 | 7.5 | 7 | 8.9 | 9.3 | 7.6 | 6.5 |
|  |  | Socializing with family/friends | 10.9 | 9.6 | 11 | 13.2 | 10.8 | 10.7 | 11.1 | 12.1 | 14.1 | 14.1 | 10.2 | 10.7 |
|  |  | Transportation | 8.7 | 7.6 | 8.2 | 7.6 | 9.2 | 8.5 | 7.9 | 6.8 | 9.2 | 8.2 | 8.3 | 7.5 |
|  |  | Hobbies | 6.8 | 7.4 | 6.1 | 5.9 | 6.8 | 7 | 6.1 | 6.2 | 7.3 | 7.2 | 6.2 | 6.5 |
|  |  | Others | 8.5 | 9.8 | 10.3 | 15.8 | 9.5 | 10.6 | 9.3 | 14.6 | 11.1 | 12.8 | 9 | 13.1 |
| Physical activity (a usual week) | | | *N* | *%* | *N* | *%* | *N* | *%* | *N* | *%* | *N* | *%* | *N* | *%* |
|  | Walk for transportation/recreation/fitness | |  |  |  |  |  |  |  |  |  |  |  |  |
|  |  | Yes | 188 | 74.6 | 182 | 71.7 | 151 | 69.3 | 219 | 76 | 70 | 70 | 300 | 73.9 |
|  |  | No | 64 | 25.4 | 72 | 28.3 | 67 | 30.7 | 69 | 24 | 30 | 30 | 106 | 26.1 |
|  | Non-walking leisure time PA | |  |  |  |  |  |  |  |  |  |  |  |  |
|  |  | Yes | 136 | 53.8 | 121 | 48 | 96 | 44 | 161 | 56.1 | 48 | 48 | 209 | 51.6 |
|  |  | No | 117 | 46.2 | 131 | 52 | 122 | 56 | 126 | 43.9 | 52 | 52 | 196 | 48.4 |
|  | Moderate intensity PA | |  |  |  |  |  |  |  |  |  |  |  |  |
|  |  | Yes | 105 | 66 | 90 | 60.4 | 68 | 60.7 | 127 | 64.8 | 34 | 63 | 161 | 63.4 |
|  |  | No | 54 | 34 | 59 | 39.6 | 44 | 39.3 | 69 | 35.2 | 20 | 37 | 93 | 26.6 |
|  | Vigorous intensity PA | |  |  |  |  |  |  |  |  |  |  |  |  |
|  |  | Yes | 55 | 35.7 | 51 | 34.9 | 37 | 33.9 | 69 | 36.1 | 17 | 32.7 | 89 | 35.9 |
|  |  | No | 99 | 64.3 | 95 | 65.1 | 72 | 66.1 | 122 | 63.9 | 35 | 67.3 | 159 | 64.1 |
|  | Confident to exercise more | |  |  |  |  |  |  |  |  |  |  |  |  |
|  |  | Not at all confident | 14 | 5.6 | 24 | 9.5 | 22 | 10 | 16 | 5.6 | 8 | 7.9 | 30 | 7.4 |
|  |  | Somewhat confident | 74 | 29.4 | 87 | 34.3 | 76 | 34.6 | 85 | 29.7 | 25 | 24.8 | 136 | 33.6 |
|  |  | Very confident | 155 | 61.5 | 133 | 52.4 | 113 | 51.4 | 175 | 61.2 | 60 | 59.4 | 228 | 56.3 |
|  | Duration (hours, a usual week)^§^ | | *M* | *SD* | *M* | *SD* | *M* | *SD* | *M* | *SD* | *M* | *SD* | *M* | *SD* |
|  |  | Walk for transportation | 3.3 | 9.9 | 3 | 7.6 | 4.5 | 10.3 | 2.4 | 8 | 4.8 | 11.2 | 2.9 | 8.5 |
|  |  | Walk for recreation/health/fitness | 4.3 | 3.7 | 4.4 | 8.9 | 4.3 | 5.4 | 4.4 | 7.6 | 4.5 | 7.3 | 4 | 3.7 |
|  |  | Moderate intensity PA | 4.2 | 4.3 | 4.4 | 5.7 | 4.3 | 5.2 | 4.3 | 4.9 | 5.6 | 5.6 | 4 | 4.8 |
|  |  | Vigorous intensity PA | 5 | 11 | 2.3 | 2.1 | 5.6 | 13.2 | 2.8 | 3.7 | 2.3 | 2.2 | 4.1 | 9 |

PA: physical activity; N: number; M: mean; SD: standard deviation; %: percentage

§Only dyads who reported “yes” to questions about whether they have smoked 100 cigarettes, participate in sedentary behaviors, or do physical activity were asked to report the degree to which they engaged in these behaviors, such as the length of time they spent doing these behaviors or packs of cigarettes they smoke.
